# Biopsies from patients with sacral insufficiency fracture are characterized by low bone matrix mineralization and high turnover

**DOI:** 10.1093/jbmrpl/ziae094

**Published:** 2024-07-12

**Authors:** Maximilian M Delsmann, Leon-Gordian Leonhardt, Assil-Ramin Alimy, Tim Hoenig, Frank Timo Beil, Klaus Püschel, Felix N von Brackel, Michael Amling, Lennart Viezens, Darius M Thiesen, Tim Rolvien

**Affiliations:** Department of Trauma and Orthopaedic Surgery, University Medical Center Hamburg-Eppendorf, 20246 Hamburg, Germany; Department of Trauma and Orthopaedic Surgery, University Medical Center Hamburg-Eppendorf, 20246 Hamburg, Germany; Department of Trauma and Orthopaedic Surgery, University Medical Center Hamburg-Eppendorf, 20246 Hamburg, Germany; Department of Trauma and Orthopaedic Surgery, University Medical Center Hamburg-Eppendorf, 20246 Hamburg, Germany; Department of Trauma and Orthopaedic Surgery, University Medical Center Hamburg-Eppendorf, 20246 Hamburg, Germany; Institute of Legal Medicine, University Medical Center Hamburg-Eppendorf, 20246 Hamburg, Germany; Institute of Osteology and Biomechanics, University Medical Center Hamburg-Eppendorf, 20246 Hamburg, Germany; Institute of Osteology and Biomechanics, University Medical Center Hamburg-Eppendorf, 20246 Hamburg, Germany; Department of Trauma and Orthopaedic Surgery, University Medical Center Hamburg-Eppendorf, 20246 Hamburg, Germany; Department of Trauma and Orthopaedic Surgery, University Medical Center Hamburg-Eppendorf, 20246 Hamburg, Germany; Department of Trauma and Orthopaedic Surgery, University Medical Center Hamburg-Eppendorf, 20246 Hamburg, Germany

**Keywords:** fracture, sacrum, bone quality, osteoporosis, osteomalacia

## Abstract

Sacral insufficiency fractures are known to occur primarily in older women without adequate trauma. While an association with low bone mineral density (ie, osteoporosis) has been reported, more detailed information on local bone quality properties in affected patients is not available. In the present study, core biopsies were obtained from the S1 sacral ala in patients with a bilateral sacral insufficiency fracture (type IV according to the fragility fractures of the pelvis classification) who required surgical stabilization. Dual energy X-ray absorptiometry (DXA) and laboratory bone metabolism analyses were performed. For comparison, control biopsies were acquired from skeletally intact age- and sex-matched donors during autopsy. A total of 31 biopsies (fracture: *n* = 19; control: *n* = 12) were evaluated by micro-computed tomography, histomorphometry on undecalcified sections, and quantitative backscattered electron imaging (qBEI). DXA measurements showed mean T-scores in the range of osteoporosis in the fracture cohort (T-score_min_ −2.6 ± 0.8). Biochemical analysis of bone metabolism parameters revealed high serum alkaline phosphatase and urinary deoxypyridinoline/creatinine levels. In the biopsies, a loss of trabecular microstructure along with increased osteoid values were detected in the fracture patients compared with controls (osteoid volume per bone volume 5.9 ± 3.5 vs. 0.9 ± 0.5%, *p* <.001). We also found evidence of microfractures with chronic healing processes (ie, microcallus) as well as pronounced hypomineralization in the biopsies of the fracture cohort compared with the controls as evidenced by lower CaMean measured by qBEI (22.5 ± 1.6 vs. 24.2 ± 0.5 wt%, *p* =.003). In conclusion, this high-resolution biopsy study provides evidence of local hypomineralization in patients with sacral insufficiency fractures, pointing to reduced fracture resistance but also a distinct phenotype other than the predominant loss of trabeculae as in postmenopausal osteoporosis. Our data highlight the importance of therapies that promote bone mineralization to optimally treat and prevent sacral insufficiency fractures.

## Introduction

Sacral insufficiency fractures are caused by repetitive microtrauma or low-energy trauma such as falls and affect mainly older patients with compromised bone status.^1^^,^^2^ These injuries may belong—at least in part—to a stress fracture-insufficiency fracture spectrum different from that of classic osteoporosis as illustrated by their increased occurrence after spinal instrumentation,^3^ total hip arthroplasty,^4^ in pregnancy and lactation,^5^ and hyperlordosis.^6^ In these conditions, weakened bones and locally increased repetitive loads may coincide, leading to the occurrence of a fracture.^7^

Sacral insufficiency fractures are included in the classification of fragility fractures of the pelvis (FFP) by Rommens and Hofmann.^8^ With the demographic changes inevitably leading to increased incidences of FFP in the western world,^9–11^ previous studies have shown that the overall impact of FFP on patient morbidity and mortality can be compared with that of proximal femur fractures.^12^^,^^13^ In clinical practice, the FFP classification has become increasingly accepted, differentiating isolated anterior or posterior pelvic injuries as well as combinations, degree of displacement, and resulting instability.^8^ Four main groups with further subcategories are described, with a higher FFP type indicating a more unstable fracture.^14^ A major difference of FFP type IV compared with type I-III fractures is the presence of spinopelvic dissociation, which often requires surgical spinopelvic fixation,^8^ unlike the possibility of conservative therapy in the other fracture types.^15^

Previous studies have focused on the analysis of local bone density in the sacrum using computed tomography (CT), demonstrating that Hounsfield units in the alar voids are lower than in the surrounding regions of the sacrum^16^ and that reduced cancellous bone density is present in the osteoporotic sacrum.^17^^,^^18^ However, CT has several limitations (eg, low resolution, lack of standardization), and studies on more detailed analyses of the sacral bone microstructure in patients with sacral insufficiency fractures are lacking. Understanding the local skeletal properties could be crucial for the prevention and treatment of these fractures to reduce their high morbidity and mortality. Therefore, the present study aims to analyze the bone microstructure and quality in specimens obtained from the sacral ala during the surgical treatment of sacral insufficiency type FFP IV fractures. We hypothesized that patients with these fractures have not only reduced bone microstructure but a specific pattern of bone-qualitative impairment, which may indicate that their pathophysiology is different from osteoporosis.

## Material and methods

### Study cohort

Patients with FFP type IV sacral insufficiency fractures (ie, spinopelvic dissociation) in whom biopsies from the sacral ala were obtained between 2021 and 2023 were assessed. In these patients, Jamshidi core needle biopsies were taken during surgery to exclude malignant bone disease, according to routine clinical procedures. Exclusion criteria were cancer, hereditary bone diseases, chronic kidney disease (glomerular filtration rate < 30 mL/min), primary hyperparathyroidism, and Paget’s disease. The biopsies were taken from a standardized region, ie, the S1 sacral ala, 2–3 cm lateral of the sacral midline, near the fracture but not in the fracture region itself. Demographic characteristics including age, sex, weight, height, and BMI were obtained from medical records and analyzed in an anonymized fashion.

A total of 31 individuals (*n* = 19 patients with sacral insufficiency fracture, *n* = 12 controls) were included in this biopsy study. Of the 21 clinical fracture biopsies originally available, two were excluded because one patient was diagnosed with osteogenesis imperfecta and the other was receiving long-term corticosteroid therapy, leaving 19 biopsies for final analysis. For comparative purposes, we collected *n* = 12 biopsies in the same region as the fracture cohort from age- and sex-matched, skeletally healthy individuals during autopsy.^19^ Circumstances leading to death were traumatic injuries or aging-associated diseases. The same exclusion criteria as for the clinical cohort were applied. The mean age of the fracture group was 77.3 ± 12.8 years and that of the control group was 83.5 ± 5.2 years (*p* =.12) (Table 1). Both cohorts consisted of mostly women (89.4% vs. 91.7%, *p* <.99). There was no difference in BMI between the studied groups (*p* = .24).

**Table 1:** Demographic overview of the fracture and control cohort.

	**Patients**	**Controls**	
	**Mean (SD) or *n* (%)**	**Mean (SD) or *n* (%)**	***p*-value**
Total (*n*)	19	12	
Age (yr)	77.3 (12.8)	83.5 (5.2)	.12
Females	17 (89.4 %)	11 (91.7 %)	>.99^#^
BMI (kg/m^2^)	23.5 (5.1)	25.7 (4.3)	.24

*n* = number of patients; yr = years; ^#^determined by the Chi^2^ test.

### **Dual-energy X-ray absorptiometry** and biochemical bone metabolism markers

Patients were examined by Dual-energy X-ray absorptiometry (DXA, Lunar iDXA, GE Healthcare). Measurements were performed at the lumbar spine and hips. T-scores expressing bone mineral density (BMD) standard deviations for young, sex-matched healthy adults were generated using the manufacturer’s software. DXA quality assurance was achieved following the institutional standard operating procedures and by daily calibration scans with a special phantom according to the manufacturer’s recommendations. Based on the T-score, the diagnosis of osteoporosis and osteopenia was made according to World Health Organization guidelines (ie, T-score ≤ −2.5).^20^ Biochemical analyses included serum levels of alkaline phosphatase (ALP), 25-hydroxyvitamin D (25-OH-D), calcium, parathyroid hormone (PTH), bone-specific ALP (bALP), osteocalcin, and urinary deoxypyridinoline (DPD)/creatinine. Measurements were performed within 4 weeks after surgery. Results were compared with reference ranges from the local laboratory for each parameter.

### Sample preparation, histology, and histomorphometry

Samples were fixed in 4% formaldehyde for at least 3 days, dehydrated, and embedded undecalcified in methyl methacrylate. Subsequently, 4-μm sections were stained with von Kossa, trichrome Goldner, and toluidine blue. Microstructural and osteoid parameters were evaluated in the histological sections using the software OsteoMeasure (OsteoMetrics, Atlanta, GA, USA) and following the guidelines of the ASBMR.^21^

### Quantitative backscattered electron imaging

After preparing histological sections, the embedded bone specimens were polished to a coplanar finish and carbon coated. Quantitative backscattered electron imaging (qBEI) was performed using a scanning electron microscope (LEO 435 VP; LEO Electron Microscopy Ltd., Cambridge, UK) with a backscattered electron detector (Type 202; K.E. Developments Ltd., Cambridge, UK).^22^^,^^23^ The scanning electron microscope was operated at 20 kV and 665 pA at a constant working distance. Four images per specimen were acquired and further analyzed using ImageJ 1.42 (National Institutes of Health, Bethesda, MD, USA)^24^ and a custom MATLAB-based program (TheMathWorks, Inc., Natick, MA, USA). As the generated gray values represent the mean calcium content (mean Ca-Wt%) of the cross-sectioned bone, the technique was used to measure the BMD distribution (BMDD). Osteocyte lacunar number and area were also analyzed from the images.

### Statistical analysis

Statistical analysis and visualizations were performed using GraphPad Prism 9 (GraphPad Software, La Jolla, CA, USA). Normal distribution of the data was tested using the Shapiro–Wilk test. For group comparisons, student’s *t*-test was performed for parametric and the Mann–Whitney test for nonparametric data. Effect sizes were calculated using Cohen’s *d* to quantify the magnitude of differences observed between the groups. For the analysis of potential associations between clinical and bone quality parameters, correlation analysis was carried out. Data are displayed as mean ± SD or as boxplot with median, interquartile range, minimum and maximum, as well as all plotted data points. All statistical tests were two-sided, with a predefined significance level set at alpha = 0.05. A post hoc power analysis was conducted using G^*^Power software v. 3.1.9.6 (Heinrich-Heine-Universität Düsseldorf, Germany) to evaluate the adequacy of the sample size used in this exploratory study. The analysis focused on the primary outcome parameters: osteoid volume/bone volume (OV/BV) and CaMean. For OV/BV, with an effect size (*d*) of 2.524 and an alpha error of 0.05, our post hoc power analysis revealed an achieved power of >0.99. For CaMean, the post hoc power analysis indicated an achieved power of 0.836. Therefore, our study had sufficient power to detect meaningful differences.

## Results

**Figure 1:**
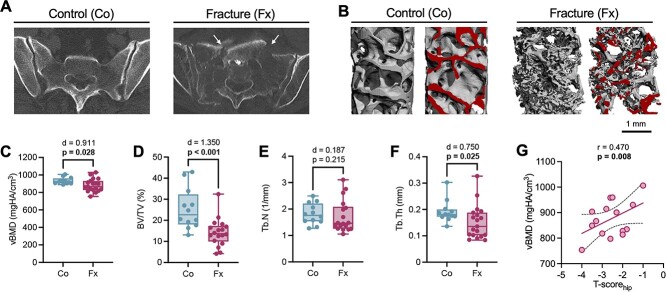
Local bone mineralization and microstructure are impaired in patients with sacral insufficiency fracture. (A) CT scans (axial view) of a representative control (Co) and fracture (Fx) case. White arrows point to the fracture line. (B) μ-CT images of core biopsies. Red color indicates virtual cut planes. (C–F) Comparison of vBMD, BV/TV, Tb.N, and Tb.Th between control and fracture groups. Data are displayed as boxplot including all data points. (G) Correlation analysis between clinical T-scores obtained at the hip and local vBMD assessed by μ-CT. Group comparisons were performed by unpaired *t*-test (normal distribution) or Mann–Whitney test. Exact *p*-values and effect sizes (Cohen’s *d*) are displayed above the panels. Numbers in bold indicate statistical significance (*p* <.05).

Representative sacral CT scans of a control and a fracture patient are shown in Figure 1A. In the fracture cohort, DXA assessment revealed low BMD with a T-score in the hip of −2.56 ± 0.82 and a lumbar T-score of −2.48 ± 1.01 (Table 2). Osteoporosis was diagnosed in 14/16 (87.5%) and osteopenia in the remaining 2/16 (12.5%) patients. In three patients, DXA examination could not be performed due to a previous multilevel spinal fusion, severe degeneration, and/or bilateral total hip arthroplasty. Due to multiple vertebral fractures, however, the criteria for clinical osteoporosis were fulfilled in these patients. Results from laboratory serum analyses showed that mean calcium (2.32 ± 0.17 mmol/L), osteocalcin (25.8 ± 8.0 μg/L), 25-OH-D (30.5 ± 9.8 μg/L), and PTH (52.8 ± 16.8 ng/L) were within the reference range. Increased values were detectable in ALP (158.9 ± 72.2 U/L) and b-ALP (32.0 ± 21.9 U/L). Finally, urinary DPD/creatinine levels were markedly increased (16.1 ± 4.1 nmol/mmol), indicating increased bone resorption in patients with sacral insufficiency fractures.

**Table 2:** DXA values and laboratory bone metabolism parameters of the fracture cohort.

**Parameter**	**Mean (SD) or *n* (%)**
*DXA*	
T-score hip (min.)	−2.56 (0.82)
T-score lumbar spine	−2.48 (1.01)
Osteoporosis (T-score ≤ −2.5)	14/16 (87.5 %)^a^
Osteopenia (T-score ≤ −1.0 > −2.5)	2/16 (12.5 %)
*Laboratory parameters*	
Calcium (2.08–2.65 mmol/l)	2.32 (0.17)
Phosphate (0.77–1.50 mmol/l)	1.07 (0.31)
ALP (46.0–116.0 U/I)	**158.9 (72.2)**
b-ALP (4.9–26.6 μg/l)	**33.4 (21.4)**
Oc (12.0–52.1 μg/l)	26.1 (7.8)
25-OH-D (>30 μg/l)	30.5 (9.5)
PTH (18.4–80.1 ng/l)	52.7 (16.2)
DPD/Crea (2.0–7.0 nmol/mmol)	**16.6 (4.3)**

Reference ranges are given in parentheses for laboratory parameters. Bold font indicates abnormal values. Abbreviation: DXA = Dual energy X-ray absorptiometry; min. = minimum; ALP = alkaline phosphatase; Oc = osteocalcin; b-ALP = bone-specific alkaline phosphatase; PTH = parathyroid hormone; DPD/Crea = deoxypyridinoline per creatinine; *n* = number of patients.

^a^In three patients, DXA measurement could not be performed at either the lumbar spine or the hip, due to the presence of implants.

Analysis of the core biopsies by micro-computed tomography (μ-CT) revealed an altered bone microarchitecture in the fracture cohort (Figure 1B). Namely, the volumetric BMD (vBMD) showed lower values in the fracture group compared with controls (control: 934.4 ± 36.0 vs. fracture: 885.7 ± 74.3 mgHA/cm^3^, *p* =.028; Figure 1C). Furthermore, lower bone volume per tissue volume (BV/TV) was observed in the fracture group compared with controls (control: 26.0 ± 1.0 vs. fracture: 18.4 ± 11.0%, *p* =.008; Figure 1D). While no differences were detected in trabecular number (Tb.N; control: 1.81 ± 0.39 vs. fracture: 2.11 ± 1.07 1/mm, *p* =.215; Figure 1E), trabecular thickness was lower in the fracture cohort (Tb.Th; control: 0.19 ± 0.04 vs. fracture: 0.16 ± 0.07 mm, *p* =.025; Figure 1F). The clinical DXA-T score measured at the hip was positively associated with localized vBMD in the sacrum (*r* = 0.47, *p* =.008, Figure 1G).

**Figure 2:**
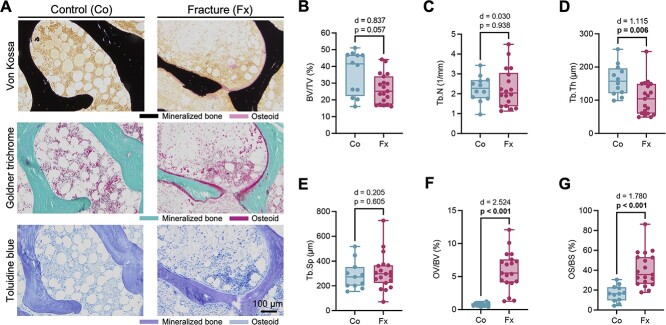
Biopsies from patients with sacral insufficiency fracture point to trabecular thinning and osteoid accumulation. (A) Histological images (von Kossa, Goldner trichrome, and toluidine blue staining; undecalcified preparation) of a representative control (Co) and fracture patient (Fx). (B–E) Comparison of BV/TV, Tb.N, and Tb.Th and Tb.Sp between biopsies obtained from controls and fracture patients. (F, G) Comparison of OV/BV and OS/BS between controls and fracture patients. Data are displayed as boxplot including all data points. Group comparisons were performed by unpaired *t*-test (normal distribution) or Mann–Whitney test. Exact *p*-values and effect sizes (Cohen’s *d*) are displayed above the panels. Numbers in bold indicate statistical significance (*p* <.05).

Visual inspection of the histological sections (Figure 2A) pointed to osteoid accumulation and increased bone turnover in the biopsies of the fracture patients. While bone volume per tissue volume (BV/TV; control 36.0 ± 12.9 vs. fracture: 27.1 ± 9.6%, *p* =.057; Figure 2B) and trabecular number (control: Tb.N; 2.25 ± 0.66 vs. fracture: 3.06 ± 1.88 1/mm, *p* =.938; Figure 2C) were not significantly different, trabecular thickness (Tb.Th; control: 160.6 ± 46.5 vs. fracture: 109.8 ± 59.3 μm, *p* =.006; Figure 2D) was lower in the biopsies of the fracture patients. No differences were observed in trabecular separation (Tb.Sp; control: 330.5 ± 191.0 vs. fracture: 321.0 ± 183.1 μm; *p* =.605; Figure 2E). Remarkably, the fracture group showed increased osteoid levels as measured by osteoid volume per bone volume (OV/BV; control: 0.88 ± 0.55 vs. fracture: 5.88 ± 3.48 %, *p* <.001; Figure 2F) and osteoid surface per bone surface (OS/BS; control: 16.4 ± 8.0 vs. fracture: 44.5 ± 20.2 %, *p* <.001; Figure 2G).

**Figure 3:**
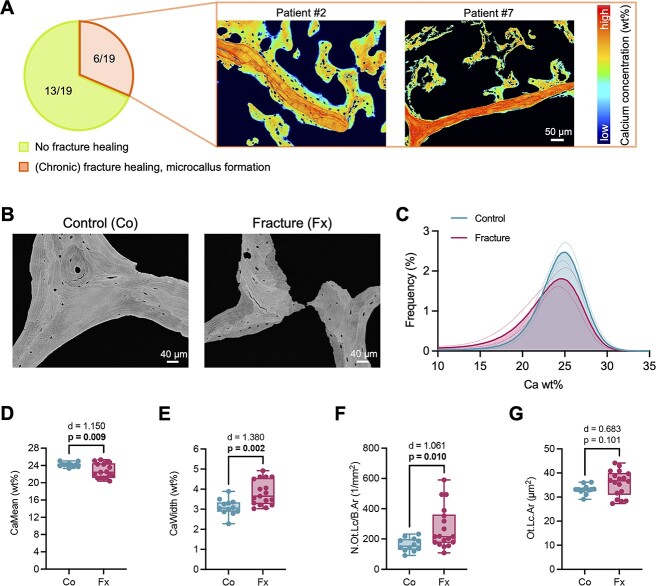
Backscattered electron microscopy identifies microfractures with signs of chronic fracture healing and impaired bone matrix mineralization. (A) Left panel: Pie charts indicating the frequency of histologically detectable signs of fracture healing. Right panel: qBEI images (pseudocolors) showing fracture healing evidenced by microcallus formation around trabeculae in two individual patients. (B) qBEI images of a representative control (Co) and fracture (Fx) case. (C) BMDD histograms. (D, E) Comparison of CaMean and CaWidth between controls and fracture patients. (F, G) Comparison of osteocyte lacunar number (N.Ot.Lc/B.Ar) and lacunar area (lc.Ar) between controls and fracture patients. Data are displayed as boxplot including all data points. Group comparisons were performed by unpaired *t*-test (normal distribution) or Mann–Whitney test. Exact *p*-values and effect sizes (Cohen’s *d*) are displayed above the panels. Numbers in bold indicate statistical significance (*p* <.05).

Although the sacral biopsies were intentionally not obtained from the fractured area as matched with preoperative CT imaging, backscattered electron images showed histologic signs of microfracture with (chronic) fracture healing processes in 6 of 19 biopsies from the fractured patients (Figure 3A). Specifically, microcallus formation around few trabeculae was found in the biopsies of individual patients. For further in-depth assessment of bone quality, this area was not considered. BMDD analysis by qBEI (Figure 3B) revealed low mean bone mineralization with greater heterogeneity in the fracture group, as visualized by a leftward shift and a broader BMDD curve in histograms of the fracture group (Figure 3C). Quantification showed lower CaMean (control: 24.2 ± 0.50 vs. fracture: 22.5 ± 1.62 wt%, *p* =.009) (Figure 3D) and higher CaWidth (control: 3.10 ± 0.40 vs. fracture: 3.85 ± 0.61 wt%, *p* =.002) (Figure 3E) values compared with controls. Evaluation of osteocyte properties revealed a higher number of osteocyte lacunae per bone area (N.Ot.Lc/B.Ar; control: 165.3 ± 27.3 vs. fracture: 289.9 ± 143.0 1/mm^2^, *p* =.010; Figure 3F) and not significantly different osteocyte lacunar area (Lc.Ar; control: 33.1 ± 1.89 μm^2^ vs. fracture: 36.9 ± 4.62, *p* =.101; Figure 3G) in the fracture cohort.

## Discussion

In this study, we investigated local bone quality characteristics in patients with sacral insufficiency fracture by applying a suite of high-resolution imaging analyses on obtained biopsies. In addition to impaired microarchitecture defined by trabecular thinning, we found a specific pattern of hypomineralization and high bone turnover consistent with clinically measured low DXA results. To our knowledge, this is the first study to investigate bone quality parameters in patients with sacral insufficiency fracture. Our findings are clinically relevant as they influence the approach to the treatment and prevention of sacral insufficiency fractures and argue for the use of treatments that improve bone mineralization instead of, or in addition to, drugs that affect only bone mass or microstructure.

The observed low DXA values were expected and are consistent with the literature.^18^ We also found a moderate correlation between the BMD T-score measured at the total hip and volumetric BMD assessed in sacral biopsies by μ-CT. However, it is known that DXA cannot distinguish between structural loss and hypomineralization (ie, osteomalacia), although the treatment for both low BMD disorders differs. Trabecular loss (ie, osteoporosis) is treated with anti-osteoporotic drugs (such as bisphosphonates and denosumab), while hypomineralization is mainly treated with vitamin D and calcium. Since mixed forms are often present and optimal effectiveness of anti-osteoporotic drugs is only guaranteed by simultaneous balancing of the calcium homeostasis, the basic treatment of osteoporosis consists of vitamin D supplementation, also due to the widespread vitamin D deficiency in western countries.^25^ Here, the skeletal mineralization defect was identified by the combined consideration of various diagnostic measures. In addition to the high ALP values,^26^^,^^27^ the abnormally high osteoid values in the histologic analysis of the undecalcified, processed specimens may also indicate osteomalacia.^25^

The qBEI analysis revealed a combination of low mean matrix mineralization and high mineralization heterogeneity, consistent with high bone remodeling. Interestingly, histologic evidence of microfracture and fracture healing was also seen, even though the biopsies were taken strictly away from the fracture site. Most likely, this indicates chronic processes affecting regions larger than the fracture area, suggesting a pathophysiology within the insufficiency fracture spectrum and different from that of osteoporosis. Clinically, this hypothesis is supported by the finding that early stages of bone insufficiency injuries manifest as bone marrow edema or bone marrow lesions on magnetic resonance imaging. Of note, patients with microdamage-induced bone marrow lesions in knee osteoarthritis showed a similar pattern of high osteoid levels, low mean matrix mineralization, and increased mineralization heterogeneity.^28^

Skeletal mineralization defects lead to impaired bone stability and reduced fracture resistance.^25^^,^^29^ Furthermore, increased mineralization heterogeneity may promote the occurrence and propagation of microcracks,^30^ which results in osteocyte apoptosis and concomitant release of nitric oxide, prostaglandin E2, and receptor activator of NF-κB ligand (RANKL).^31^^,^^32^ These factors activate osteoclasts, resulting in increased bone resorption.^33^^,^^34^ While these considerations link poor bone mineralization to bone loss, we also identified a component of bone loss (ie, osteoporosis) in our patients that manifested as lower bone volume fraction and elevated laboratory resorption markers such as DPD.^35^

Another insight from our analysis was the tendency toward enlarged osteocyte lacunae in biopsies of the fracture patients compared with controls. An enlargement of osteocyte lacunae has already been demonstrated as a reversible phenomenon in lactation^36^ and vitamin D deficiency^37^ to release calcium from skeletal stores, also referred to as osteocytic osteolysis or perilacunar/canalicular remodeling. Postpartum sacral insufficiency fractures are a well-known entity but whether findings are comparable to geriatric patients remains to be determined in future studies. Importantly however, these alterations in osteocyte properties may additionally contribute to reduced fracture resistance.^38^ Of note, the observed increased number of osteocyte lacunae is most likely a consequence of increased bone remodeling.

The fact that serum calcium, phosphate, and vitamin D levels were normal in most patients does not necessarily imply that calcium homeostasis is intact and, in fact, vitamin D substitution had already been initiated in most patients prior to laboratory measurement. The reasons for impaired calcium homeostasis are multifaceted and include impaired enteral calcium absorption due to lack of gastric acid (ie, hypochlorhydria).^39^ Hypochlorhydria is common in the elderly and can be caused by chronic use of proton pump inhibitors, leading to compromised calcium absorption.^40^ In these patients, high ALP and high PTH levels are often the only evidence of impaired calcium homeostasis, as the organism maintains normal calcium levels by activating bone resorption. Importantly, only specific calcium supplements, such as calcium gluconate, have been shown to be effective to balance calcium homeostasis.^41^ Therefore, these supplements should be considered in affected patients.

Our study has some limitations. The relatively small number of included biopsies may limit generalizability. Nonetheless, our high-resolution workup provides unique insights into the pathophysiology of sacral insufficiency fractures. Another limitation is the unknown extent to which fracture repair mechanisms and immobilization due to pain may have influenced the results of histologic analysis. In addition, the biopsy taken from the bone affected by the fracture may not be representative of the whole skeleton. However, to address this issue, biopsies were taken strictly near the fracture but not in the fracture region itself. A further limitation of our study is that the laboratory assessment was usually initiated after surgery, which could be the reason for higher vitamin D levels than expected for this cohort, as vitamin D supplementation was started at the latest after diagnosis. Furthermore, only FFP type IV fractures could be included. However, since most fracture types are treated conservatively, biopsy material was not available for these. Finally, a further limitation arises from the fact that this is a cross-sectional study, which is associated with inherent limitations, including the fact that no causal inferences can be made.

Taken together, patients with sacral insufficiency fractures are characterized by a distinct mineralization defect that is more pronounced than impaired bone microstructure. Our study highlights the importance of therapies that promote bone mineralization to prevent sacral insufficiency fractures and to treat fractures that have occurred, for instance through compensation of impaired calcium homeostasis by vitamin D and/or calcium supplementation. A possible benefit of such basic therapy should be investigated in future studies.

## Data Availability

The data that support the findings of this study are available from the corresponding author upon reasonable request.
